# The fate of the new pharmacy bill: going backwards or forwards?

**DOI:** 10.1186/s40545-016-0081-7

**Published:** 2016-09-22

**Authors:** Kah Seng Lee, Yen Wei Lim, Long Chiau Ming

**Affiliations:** 1 Unit for Medication Outcomes Research and Education (UMORE), Pharmacy, School of Medicine, University of Tasmania, 7001 Hobart, Tasmania Australia; 2 School of Pharmaceutical Sciences, Universiti Sains Malaysia, 11800 Gelugor, Penang Malaysia; 3 Vector-borne Diseases Research Group (VERDI), Pharmaceutical and Life Sciences Community of Research, Faculty ofPharmacy, Universiti Teknologi MARA, 42300 Puncak Alam, Selangor Malaysia

**Keywords:** Governance for medicine, Consumer engagement, Transparency, Public debate, Dispensing separation

## Abstract

**Background:**

The proposed Pharmacy Bill of Malaysia which served to consolidate and harmonise the existing pharmacy legislation which has been used for more than 60 years. This new Pharmacy Bill contains 17 parts and a total of 170 legislative sections covering laws governing pharmacy practice, medicinal products classification, registration, sale, supply, licensing etc. Our article could serve as a case study on pharmacy jurisprudence and drug regulation as well as the governance for medicines.

**Discussion:**

Changes to the colonial era legislation are long overdue as the present pharmaceutical and medical controls are not integrated and various overlaps exist in terms of roles of control. However, various organisations of private general practitioners strongly opposed this Pharmacy Bill and lobbied for a revised version that greatly favours themselves. Thus, the latest revision of this Pharmacy Bill renders the power to medical doctors to not only continue selling and supplying medications but also compound medication.

**Summary:**

A complete overhaul of pharmacy legislation in view of the current challenges faced in providing efficient and comprehensive health services in Malaysia is necessary. For the sake of patients’ safety and good governance for medicines, the private general practitioners should empower the patients with their needs for prescription and itemised billing. The proposed Pharmacy Bill could make the whole mechanism of managing and controlling the use of medicines more transparent and synchronised.

## Background

A New Pharmacy Bill (NPB) is expected to be tabled in the Malaysian Parliament this year after obtaining approval from the Attorney General. The current pharmacy legislation is made up of separate acts, namely the Poisons Act (1952), Sale of Drugs Act (1952), Registration of Pharmacists Act (1951) and the Medicines (Advertisement and Sale) Act (1956) [1]. The consolidation of these pre-independence laws into the NPB is expected to address lacunae in the existing legislation and safeguard public health and safety by harmonising the classification of drugs, and providing stronger deterrent penalties for offences regarding counterfeit medicinal products, psychotropic distribution and the diversion of precursor chemicals [2]. Notwithstanding this, the proposed NPB has been strongly opposed by the various medical associations and federations in Malaysia on the grounds that provisions in the legislation have the potential to deprive doctors of the legal right to sell and supply medicine to patients [3, 4]. In our view, this negative reaction is mainly due to a misinterpretation of the draft content, and that, in reality, the NPB does not include any terms on dispensing separation.

To formalise the discussion, Federation of Private Medical Associations Malaysia organised a National Forum on the NPB and Relevant Laws, held in April 2015. Subsequently, 17 associations, mainly representing private medical practitioners from various states in Malaysia, presented a joint memorandum to request the Health Ministry to cancel numerous laws that were deemed disadvantageous to their practice. On a similar note, the Malaysian Medical Association rejected the NPB, complaining that relevant stakeholders had not been consulted, and that the bill had been brought forward without proper consideration of the privileges of these stakeholders, especially in respect to the sale and supply of medicine by doctors.

### Are we reversing health care system back to colonial era?

The Poisons Act currently regulates the importation, possession, manufacturing, compounding, storage, transportation, sale and use of poisons by doctors, dentists, veterinarians, manufacturers, distributors and retail stores. At present, scheduled poisons (in layman’s term medicines), according to the Poisons Act, are mainly grouped as Groups A, B and C poisons, as well as psychotropic drugs/substances (drug that changes brain function and alter perception, mood and consciousness). Under this classification system, Group A poisons cannot be used in humans, but only for trade or scientific research purposes. Group B poisons and psychotropic drugs can be supplied with a prescription by a physician, dentist, veterinarian or pharmacist; while Group C poisons can be supplied without a prescription by a pharmacist holding a valid practising license. In the proposed NPB, poisons will be categorised into three categories, namely prescription only medicines (POM), pharmacy medicines (PM) and general sales list medicines (GSL).

- POMs cover psychotropic and narcotic medical products, and medical products formerly classified as Group B poisons that required a prescription to be supplied.
- PMs can be obtained from licensed pharmacists without a prescription. These consist of psychotropically active substances and precursors formerly in the Group B and C poison categories. PMs also include finished products containing drugs such as antihistamine, pholcodine, antidiabetics, and external preparations containing an antibiotic or steroid.
- GSL consists of registered products such as traditional medicines or supplements or cosmetics which are available over-the-counter.

Advocates of the proposed NPB hailed the reclassification of medicines as necessary and timely since the current scheduled poison system is confusing and outdated. The general public does not understand and know the differences between medicines in Group B and C. Unintentionally, patients sometimes request Group B medicines from a pharmacy without a proper prescription. With the new classification of PM, a clear label of “pharmacy medicine” could be printed on the product packaging to indicate that the medicine in question could be supplied by a licensed pharmacist.

The proposed classification is not uncommon, with the United Kingdom, Singapore and many other countries having adopting such a system. It provides a clearer description of the medicines group and the basis on which these can be supplied to patients. Some medicines are in dire need of reclassification since inappropriate or outdated classifications are currently restricting the availability and affordability of these medicines. For instance, the Singapore Health Sciences Authority, in October 2015, declassified cetirizine (an antihistamine to treat flu and allergic reactions) from PM to GSL [5], while in Malaysia, it remains classified as a Group C poison, only to be sold by a pharmacist. The reclassification of active psychotropic substances and precursors formerly in the Group B poison category to the PM group has also been grossly misinterpreted by certain medical associations [3], who argue that psychotropic drugs will now be classified PM, whereas currently doctors are allowed to sell and supply psychotropic drugs without screening by a pharmacist. It must be highlighted that active psychotropic substances and precursors are actually the raw active pharmaceutical ingredients (raw powder) of psychotropic drugs that are used in the manufacturing of pharmaceutical dosage forms (tablets, capsule and syrup) or for import or export purposes. Under the NPB, doctors are still allowed to sale and supple psychotropic drugs such as buprenorphine tablet or methadone syrup.

This oversight led to a joint memorandum being proposed in respect to the NPB by various medical and dental organisations, arguing that the new classification of “Pharmacist Medicines” will result in an effective monopoly being created on behalf of pharmacists in respect to the supply of medicines, and including psychotropic drugs that are currently listed as Group B poisons [3]. A further concerned expressed in the joint memorandum is that the Senior Director of Pharmaceutical Services is viewed as having a free hand to classify medicines based on profession, effectively taking away, or severely curtailing, the dispensing role of medical practitioner, dental practitioners and veterinary surgeons in private facilities. Reluctant to countenance any changes, opponents of the NPB are insisting on maintaining status quo in pharmacy legislation, arguing that the existing system is a proven affordable and convenient system of care since the doctors could provide diagnosis and medication independently.

From another viewpoint, the Secretary General of the Malaysian Consumers’ Association has stressed the importance of empowering consumers with choices so they can make the best decisions according to their needs. The concern is not who dispenses the medicine but whether the patients are getting the maximum benefits from the changes in legislation. Medication consultation and screening performed by pharmacists not only minimise the prescription error but also ensure patients are counselled correctly in term of potential drug interactions and dosing. Another worrying trend seem in Malaysia is private medical practitioners tend to prescribe seven times more medicines which might not be essential for the patient’s condition [6]. Over-prescribing of non-essential medication will further burden the patient and jeopardize patient’s safety since number of medication prescribed is a predictor for medication error [7].

### Dispensing doctors lead to uncontrolled medication prices?

Another concern often raised by public is non-itemised billing by the general practitioner (GP) and private hospitals. The associations of general practitioner tend to cite the reason that GP compensate the consultation fees on the medicine prices. This ambiguous charging system might not be suitable now because generic medicines are widely available now. A generic medicine could easily cost 3–6 times less than the innovator product. For example a box (30 tablets) of generic losartan tablet for treatment of hypertension could cost MYR 15 (equivalent to USD 3.75) (USD 1 ≈ MYR 4, IMF 2016) [8] compare to the innovator of MYR 83.40 (USD 20.85). Furthermore, a box (30 tablets) of generic clopidogrel tablet for blood thinning could cost RM 70 (USD 17.50) compare to the innovator (14 tablets) of RM 90 (USD 22.50). Tested and proven health care financing system in countries such as Korea, Australia and Taiwan have switched to pharmacy dispensing to facilitate medicine pricing regulation. Coming back to Malaysia scenario, currently there are no relevant laws that give authority to the pharmacy enforcement officer to control drug prices sold in this country. In order to curb unregulated medicine pricing in private sector, the Health Minister stated that the proposed NPB would include legitimate mechanisms for medicine pricing regulation. The effort to regulate medicine prices is a step in the right direction since there is currently a lack of transparency in pharmaceutical supply chains. Price disparities exist within and between distribution channels. Unfair bonuses, discounts and rebates are examples of the tactics employed by pharmaceutical companies to distort demand, thus creating an unhealthy and dysfunctional market and business environment. GPs, as the primary initiators for pharmaceutical demand, are given better prices compared to private pharmacies. Although preventive measure have been taken by the Malaysia’s Ministry of Health issuing a Guideline on Good Pharmaceutical Trade Practice, its effectiveness is doubtful as it has no legally binding [9]. It must be highlighted that a lower cost price given to GPs has not translated into a cheaper drug price as the patients are still charged the recommended retail price. Therefore, pharmacy is considered as the preferred choice for the treatment of minor ailments whereby a patient could purchase two to three types of medicines with approximately RM10 (USD 2.50) in a pharmacy as compared to more than RM40 (USD 10) in a GP clinic.

## Conclusions

Changes to the colonial era legislation are long overdue as the present pharmaceutical and medical controls are not integrated and various overlaps exist in terms of roles of control. These changes could make the whole mechanism of managing and controlling the use of medicines more transparent and synchronised. The work on the proposed NPB has been on the drawing board for the past 20 years, and the time is now right for a complete overhaul of pharmacy legislation in view of the current challenges faced in providing efficient and comprehensive health services in Malaysia.

## Abbreviations

GP: General practitioner
GSL: General sales list medicines
NPB: New pharmacy bill
PM: Pharmacy medicines
POM: Prescription only medicines
USD: United states dollar
